# Molecular subtypes predict the preferential site of distant metastasis in advanced breast cancer: a nationwide retrospective study

**DOI:** 10.3389/fonc.2023.978985

**Published:** 2023-01-25

**Authors:** Jin-Hu Fan, Su Zhang, Huan Yang, Zong-Bi Yi, Qu-Chang Ouyang, Min Yan, Xiao-Jia Wang, Xi-Chun Hu, Ze-Fei Jiang, Tao Huang, Zhong-Sheng Tong, Shu-Sen Wang, Yong-Mei Yin, Hui Li, Run-Xiang Yang, Hua-Wei Yang, Yue-E. Teng, Tao Sun, Li Cai, Hong-Yuan Li, Xue-Nong Ouyang, Jian-Jun He, Xin-Lan Liu, Shun-E. Yang, Jia-Yu Wang, Bing-He Xu, You-Lin Qiao

**Affiliations:** ^1^ Department of Cancer Epidemiology, National Cancer Center/National Clinical Research Center for Cancer/Cancer Hospital, Chinese Academy of Medical Sciences and Peking Union Medical College, Beijing, China; ^2^ Department of Operations Management, The First Affiliated Hospital of Dalian Medical University, Dalian, China; ^3^ Department of Medical Oncology, National Cancer Center/National Clinical Research Center for Cancer/Cancer Hospital, Chinese Academy of Medical Sciences and Peking Union Medical College, Beijing, China; ^4^ Department of Breast Cancer Medical Oncology, Hunan Cancer Hospital, Changsha, China; ^5^ Department of Breast Disease, Henan Breast Cancer Center, The affiliated Cancer Hospital of Zhengzhou University & Henan Cancer Hospital, Zhengzhou, China; ^6^ Department of Medical Oncology, Zhejiang Cancer Hospital, Hangzhou, China; ^7^ Department of Medical Oncology, Fudan University Shanghai Cancer Center, Shanghai, China; ^8^ Department of Breast Cancer, The Fifth Medical Centre of Chinese People’s Liberation Army (PLA) General Hospital, Beijing, China; ^9^ Department of Breast and Thyroid Surgery, Union Hospital, Tongji Medical College, Huazhong University of Science and Technology, Wuhan, China; ^10^ Department of Breast Oncology, Key Laboratory of Breast Cancer Prevention and Therapy, National Clinical Research Center for Cancer, Tianjin Medical University Cancer Institute and Hospital, Tianjin, China; ^11^ Department of Medical Oncology, State Key Laboratory of Oncology in South China, Sun Yat-sen University Cancer Center, Guangzhou, China; ^12^ Department of Medical Oncology, The First Affiliated Hospital of Nanjing Medical University, Nanjing, China; ^13^ Department of Breast Surgery, Sichuan Province Tumor Hospital, Chengdu, Sichuan, China; ^14^ Department of Medical Oncology, Yunnan Cancer Hospital, Kunming Medical University, Kunming, China; ^15^ Department of Breast Surgery, The Affiliated Tumor Hospital of Guangxi Medical University, Nanning, China; ^16^ Departments of Medical Oncology and Thoracic Surgery, The First Hospital of China Medical University, Shenyang, China; ^17^ Department of Medical Oncology, Cancer Hospital of China Medical University, Liaoning Cancer Hospital and Institute, Key Laboratory of Liaoning Breast Cancer Research, Shenyang, China; ^18^ The 4th Department of Internal Medical Oncology, Harbin Medical University Cancer Hospital, Harbin, China; ^19^ Department of the Endocrine and Breast Surgery, The First Affiliated Hospital of Chongqing Medical University, Chongqing Medical University, Chongqing, China; ^20^ Department of Medicine Oncology, 900Hospital of the Joint Logistics Team, Fuzhou, China; ^21^ Department of Breast Surgery, The First Affiliated Hospital of Xi’an Jiaotong University, Xi’an, China; ^22^ Department of oncology, General Hospital of Ningxia Medical University, Ningxia, China; ^23^ Department of Breast Cancer and Lymphoma, Affiliated Tumor Hospital of Xinjiang Medical University, Urumqi, China

**Keywords:** advanced breast cancer, molecular subtype, site of distant metastasis, retrospective study, clinical epidemiological characteristic

## Abstract

**Objective:**

This study aimed to explore possible associations between molecular subtypes and site of distant metastasis in advanced breast cancer (ABC).

**Methods:**

3577 ABC patients were selected from 21 hospitals of seven geographic regions in China from 2012-2014. A questionnaire was designed to collect medical information regarding demographic characteristics, risk factors, molecular subtype, recurrence/metastasis information, and disease-free survival (DFS). The cancers were classified into Luminal A, Luminal B, HER2-enriched and Triple Negative subtypes. Chi-square test and multivariate Cox proportional hazard models were performed to explore the associations between molecular subtypes and distant metastasis sites.

**Results:**

A total of 2393 cases with molecular subtypes information were finally examined. Patients with Luminal A (51.1%) and Luminal B (44.7%) were most prone to bone metastasis, whereas liver metastasis was more frequently observed in HER2-enriched ABC patients (29.1%).The cumulative recurrence and metastasis rates of ABC patients at 36 months of DFS were the most significant within molecular types, of which Triple Negative was the highest (82.7%), while that of Luminal A was the lowest (58.4%). In the adjusted Cox regression analysis, Luminal B, HER2-enriched and Triple Negative subtypes increased the risk of visceral metastasis by 23%, 46% and 87% respectively. In addition, Triple Negative patients had a higher probability of brain metastasis (HR 3.07, 95% CI: 1.04-9.07).

**Conclusion:**

Molecular subtypes can predict the preferential sites of distant metastasis, emphasizing that these associations were of great help in choices for surveillance, developing appropriate screening and cancer management strategies for follow-up and personalized therapy in ABC patients.

## Introduction

Breast cancer is the most common malignancy in women worldwide. Approximately 2,261,419 new cases and 416,371 deaths occurred in 2020 (1). Although the prognosis of breast cancer patients is generally favorable and mortality has declined due to early detection, optimal surgery and improved adjuvant therapies, 20%–30% of patients will still develop distant metastases and cases with progressive stage only have a median two-year survival time (2, 3). The distant organs to which breast cancer preferentially metastasizes, of which bone, liver, lung, and brain are among the most common sites, are associated with the patients’ survival outcomes (4, 5). Patients with brain metastases or metastases at multiple sites generally have relatively poor prognostic outcome compared with lung and other visceral metastases (6). Breast cancer is no longer seen as a single disease but rather a multifaceted disease comprised of distinct biological subtypes with diverse natural history, presenting a varied spectrum of clinical, pathologic and molecular features with different prognostic and therapeutic implications.

Breast cancer is widely recognized as a heterogeneous disease with a variable clinical and pathological behavior and also different prognosis and response to cancer therapies, which makes it difficult not only to define prognosis of this disease, but also predict the risk of metastasis (7, 8). It has long been accepted that common risk factors such as tumor size, histologic grade, clinical stage and hormone receptor status have with increasing importance influenced the progression of malignancy and the pattern of distant metastasis (9, 10). Also, molecular subtype is being increasingly considered as an indicator to further reveal distinct characteristics and mechanisms for different organ-specific metastatic breast cancer variants (11). Four major molecular subtypes of breast cancer have been identified based on their gene expression profiling and immunohistochemical results: Luminal A, Luminal B, HER2-enriched and Triple Negative. There is growing evidence indicating that patterns of metastasis and clinical outcome are different between different subtypes: Luminal tumors are associated with a better prognosis compared with HER2-enriched or Triple negative tumors which have a more aggressive clinical outcome (12, 13). Our research team has already conducted a 10-year retrospective multi-center study of breast cancer in China (14). However, data are limited concerning advanced breast cancer (ABC) at a national level. Therefore, in the present study, we sought to investigate the possible relationship between molecular subtypes and the preferential distant metastasis sites among ABC patients to guide treatment decision making and develop appropriate surveillance strategies.

## Materials and methods

### Institutional review board

This study was approved by the Ethics Committee of Cancer Hospital, Chinese Academy of Medical Sciences (CHCAMS). Patient consent was not required as there were no risks anticipated to the participants of the study. All data were stripped of any patient identifiers.

### Study design and hospital selection

This study was a 3-year retrospective multi-center analysis in China from January 1st, 2012 to December 31st, 2014. Female ABC patients were selected randomly from 21 tertiary hospitals of seven geographic regions. Traditionally, China was divided into seven geographical regions: north, northeast, central, south, east, northwest, and southwest, three tertiary hospitals were selected from each region randomly. The aim of this method was to establish a large group of participants in ABC with complete pathological diagnosis and treatment information, which could fully represent the differences between urban and rural areas, including women of different living habits and socio-economic status in different geographical regions.

### Patients and inclusion criteria

The subjects of this study were pathologically confirmed ABC patients with recurrence and distant metastasis hospitalized within the specified period. The number of collected cases were determined proportionally according to the inherent population capacity of each geographical region. In each hospital, one month was randomly selected for each year according to the random number and all pathologically diagnosed ABC cases for that month were reviewed. January and February were excluded from the random selection to decrease any confounding effects of the Chinese New Year holiday (the longest holiday period of the year). Firstly, all eligible cases were recruited in 2012, if inpatients admissions were less than predetermined numbers, more cases from the neighboring months were reviewed in that year, otherwise, the remaining cases continued to be collected in 2013 and 2014, until the total number in that year reaches the target quantity.

All patients enrolled in this study must meet 3 key inclusion criteria: (1) pathology confirmed female ABC, when diagnosed as stage IV or local recurrence/distant metastasis after diagnosis; (2) inpatient admission date was within the selected month in the study hospital and (3) full information of surgical pathology results, time and location of metastasis, detailed therapeutic methods and protocols after metastasis.

### Data collection and quality control

Medical records were reviewed by local clerks within each hospital according to the designated protocol and a questionnaire was designed by epidemiologists and breast physicians in CHCAMS to collect information from each enrolled patient’s chart regarding (1) General information; (2) Demographic characteristics and breast cancer risk factors; (3) Clinical and imaging diagnostic information at first diagnosis; (4) Molecular subtype of patients; (5) Surgical and adjuvant treatment information; (6) Recurrence and metastasis treatment information including distant metastasis sites, disease-free survival (DFS), endocrine therapy, targeted therapy and chemotherapy after metastasis.

Two data-input clerks from each hospital were recruited to independently double-enter all above information from the paper to computer-based database. Then all finished double-entry databases were sent to CHCAMS for validation by running EpiData. Inconsistencies between the two databases revealed by CHCAMS were reported to the local clerks for adjudication until the databases were consistent. As a final inspection, one of the databases was chosen to undergo a final consistency check. Logical errors (e.g. the first recurrence and metastasis date were earlier than the date of the first diagnosis of breast cancer) were reported back to the local hospital, and the local collaborators reviewed the original medical chart again. After checking with the original medical record, the local staff sent the revised database back to CHCAMS for a final analysis. During the consistency check, 5% of the medical charts were randomly selected based on the study ID and sent to CHCAMS for quality control review.

### Molecular subtypes

The cancers were classified into four molecular subtypes according to the St-gallen Guidelines, 2013 (15): (1) Luminal A: ER+, PR+, HER2- and Ki-67<14%; (2) Luminal B: ① ER+, HER2- and at least one of: Ki-67 > 14%, PR- or low expression, low recurrence risk based on polygene expression analysis (if applicable); ② ER+, HER2+, any Ki-67 and any PR; (3) HER2-enriched: ER-, PR- and HER2+; (4) triple negative: ER-, PR- and HER2-.

### Recurrence and distant metastasis

Recurrence and distant metastasis were diagnosed by clinical evaluations including imaging studies or biopsy. Distant metastasis was defined as metastasis of breast cancer developing beyond the ipsilateral or contralateral breast, chest wall, or regional lymph node including ipsilateral axillary, supraclavicular, or internal mammary lymph node. Five patterns of the distant metastasis were mainly classified: bone metastasis only, metastases excluding bone and brain, bone metastasis + others (excluding brain metastasis), brain metastasis only and brain metastasis + others. Cumulative frequency of bone, liver, lung, brain and visceral metastases (including liver, lung, brain, adrenal gland, ovary, etc.) in this study was analyzed regarding to the molecular subtypes. Disease free survival (DFS) was defined as the time from the first diagnosis of breast cancer by surgery or puncture to recurrence and/or metastasis, whichever was the earliest.

### Statistical analysis

Patients’ demographic and clinicopathological characteristics, pattern of the distant metastasis and cumulative frequency of the metastasis sites were compared within subtypes using chi-square (χ^2^) test or fisher for categorical variables as appropriate and analysis of variance (ANOVA) for continuous variables. The Kaplan-Meier analysis was used for cumulative recurrence and metastasis rates with a log-rank test to assess the significance among the four molecular subtypes. Cox proportional hazards regression models were used to determine hazard ratios (HRs) and 95% confidence intervals (Cis) for the associations between molecular subtypes and the risk of distant metastasis at specific sites. Potential confounders included age at diagnosis, tumor size, number of lymph node metastasis, menstrual status, education and family history. In the pairwise comparisons, Luminal A was set as the referral group for the better prognosis, all other subtypes compared with it. Statistical significance was assessed using two-tailed tests with a significant level of 0.05. Analyses were conducted using SPSS statistical software version 22.0 (IBM Corp, Armonk, NY, http://www.ibm.com).

## Results

### Clinicopathological characteristics

A total of 3577 female patients with pathologically confirmed ABC from January 1st, 2012 to December 31st, 2014 were identified. 1184 patients were excluded from analysis with missing information of molecular subtypes. Therefore, 2393 patients included in the final analysis. The most common subtype in ABC patients was Luminal types (1361/2393, 56.9%), followed by HER2-enriched (523/2393, 21.9%), Triple Negative (509/2393, 21.3%). Clinicopathological characteristics of ABC patients according to molecular subtypes are shown in **Table 1**. Age at diagnosis, household register, education, surgery, adjuvant chemotherapy, adjuvant endocrine therapy and DFS were found to be significantly different among the four molecular subtypes (P<0.05). The subtype of Triple negative was older at first diagnosis and rural household registration, was more likely to have lower level of education and a relatively shorter DFS, compared with other three subtypes.

**Table 1:** Clinicopathological characteristics of ABC patients according to molecular subtypes.

	Total	Luminal A	Luminal B	HER2-enriched	Triple Negative	P value
Age at diagnosis (years)						
Mean ± SD	46.51 ± 10.40	45.70 ± 10.53	45.86 ± 10.47	47.19 ± 10.71	47.65 ± 9.69	0.002
≤45	1125 (47.0%)	182 (52.3%)	504 (49.8%)	240 (45.9%)	199 (39.1%)	<0.001
>45	1268 (53.0%)	166 (47.7%)	509 (50.2%)	283 (54.1%)	310 (60.9%)	
BMI (Kg/m^2^)						
Mean ± SD	23.89 ± 3.37	23.96 ± 3.42	23.68 ± 3.33	24.09 ± 3.19	24.07 ± 3.58	0.083
Underweight (≤18.49)	87 (4.1%)	9 (2.9%)	42 (4.6%)	15 (3.2%)	21 (4.6%)	0.479
Normal Weight (18.50~24.99)	1313 (61.5%)	194 (62.2%)	573 (63.4%)	276 (59.7%)	270 (59.2%)	
Overweight (25.00~29.99)	626 (29.3%)	93 (29.8%)	246 (27.2%)	150 (32.5%)	137 (30.0%)	
Obesity (>29.99)	108 (5.1%)	16 (5.1%)	43 (4.8%)	21(4.5%)	28 (6.1%)	
Household register						
Urban	1146 (48.1%)	152 (44.1%)	490 (48.4%)	269 (51.6%)	235 (46.4%)	0.010
Rural	872 (36.6%)	123 (35.7%)	375 (37.1%)	167 (32.1%)	207 (40.8%)	
Unknown	367 (15.4%)	70(20.3%)	147(14.5%)	85 (16.3%)	65 (12.8%)	
Education						
≤Primary school education	244 (10.2%)	33 (9.6%)	110 (10.9%)	42 (8.0%)	59 (11.7%)	0.005
Middle school education	191 (8.0%)	17 (5.0%)	88 (8.7%)	37 (7.1%)	49 (9.7%)	
≥ High school education	276 (11.6%)	33 (9.6%)	135 (13.4%)	48 (9.2%)	60 (11.9%)	
Unknown	1670 (70.1%)	260(75.8%)	677 (67.0%)	395 (75.7%)	338 (66.8%)	
Marital status (%)						
Unmarried	73 (3.1%)	9 (2.6%)	32 (3.2%)	18 (3.5%)	14 (2.8%)	0.983
Married	2269 (95.7%)	328 (96.2%)	959 (95.6%)	496 (95.6%)	486 (95.9%)	
Widowed/divorced	28 (1.2%)	4 (1.2%)	12 (1.2%)	5 (1.0%)	7 (1.4%)	
Menstrual status (%)						
Premenopausal	1097 (49.1%)	163 (50.3%)	477 (50.3%)	246 (50.4%)	211 (44.5%)	0.170
Postmenopausal	1138 (50.9%)	161 (49.7%)	47 2(49.7%)	242 (49.6%)	263(55.5%)	
Family history						
Yes	111 (4.9%)	14 (4.2%)	48 (5.0%)	30 (6.0%)	19 (3.9%)	0.442
No	2162 (95.1%)	317 (95.8%)	914 (95.0%)	467 (94.0%)	464 (96.1%)	
Smoking status						
Yes	26 (1.2%)	4 (1.3%)	7 (0.8%)	7 (1.5%)	8 (1.7%)	0.415
No	2143 (98.8%)	308 (98.7%)	908 (99.2%)	473 (98.5%)	454 (98.3%)	
Drinking status						
Yes	36 (1.7%)	5 (1.6%)	15 (1.6%)	9 (1.9%)	7 (1.5%)	0.976
No	2133 (98.3%)	307 (98.4%)	900 (98.4%)	470 (98.1%)	456 (98.5%)	
Tumor size (cm)						
≤ 2	440 (26.0%)	80 (31.7%)	179 (25.4%)	95 (25.9%%)	86 (23.1%)	0.220
2-5	986 (58.2%)	138 (54.8%)	415 (58.9%)	217 (59.1%)	216 (58.1%)	
>5	269 (15.9%)	34 (13.5%)	110 (15.6%)	55 (15.0%)	70 (18.8%)	
Number of lymph node metastasis						
0	849 (35.5%)	120 (34.5%)	345 (34.1%)	202 (38.6%)	182 (35.8%)	0.188
1-3	611 (25.5%)	103 (29.6%)	255 (25.2%)	138 (26.4%)	115 (22.6%)	
3-9	485 (20.3%)	70 (20.1%)	207 (20.4%)	97 (18.5%)	111 (21.8%)	
>9	448 (18.7%)	55 (15.8%)	206 (20.3%)	86 (16.4%)	101 (19.8%)	
Pathological type (%)						
Carcinoma in situ	57 (2.4%)	6 (1.8%)	18 (1.8%)	16 (3.1%)	17 (3.4%)	0.102
Invasive ductal carcinoma	1877 (80.4%)	265 (79.3%)	795 (80.3%)	415 (81.2%)	402 (80.2%)	
Other invasive carcinoma	254 (10.9%)	47 (14.1%)	116 (11.7%)	43 (8.4%)	48 (9.6%)	
Others	148 (6.3%)	16 (4.8%)	61 (6.2%)	37 (7.2%)	34 (6.8%)	
Surgery						
Yes	2155 (90.5%)	316 (92.1%)	890 (88.1%)	495 (94.8%)	454 (89.7%)	<0.001
No	223 (9.4%)	27 (7.9%)	119 (11.8%)	25 (4.8%)	52 (10.3%)	
Surgical method						
Mastectomy	1902 (90.6%)	278 (92.4%)	774 (88.9%)	440 (90.3%)	410 (93.2%)	0.053
Conservative surgery	197 (9.4%)	23 (7.6%)	97 (11.1%)	47 (9.7%)	30 (6.8%)	
Adjuvant chemotherapy						
Yes	1930 (82.8%)	271 (80.4%)	804 (81.5%)	454 (88.5%)	401 (81.0%)	0.002
No	402 (17.2%)	66 (19.6%)	183 (18.5%)	59 (11.5%)	94 (19.0%)	
Adjuvant radiotherapy						
Yes	960 (41.6%)	128 (38.6%)	412 (42.3%)	226 (44.7%)	194 (39.2%)	0.205
No	1348 (58.4%)	204 (61.4%)	563 (57.7%)	280 (55.3%)	301 (60.8%)	
Adjuvant endocrine therapy						
Yes	923 (39.6%)	212 (63.7%)	610 (61.7%)	42 (8.3%)	59 (11.8%)	<0.001
No	1406 (60.4)	121 (36.3%)	379 (38.3%)	466 (91.7%)	440 (88.2%)	
DFS (months)						
Median	29.0	36.0	27.0	20.0	18.5	<0.001
≤24	998 (50.4%)	86 (30.7%)	369 (45.4%)	286 (60.2%)	257 (62.1%)	<0.001
>24	984 (49.6%)	194 (69.3%)	444 (54.6%)	189 (39.8%)	157 (37.9%)	

### Pattern of distant metastasis and molecular subtypes

The difference of the pattern of distant metastasis was significantly different among subtypes (P<0.001, **Table 2**). The pattern of bone metastasis only was more frequently observed in Luminal A and Luminal B subtypes (24.4% and 18.9%, respectively), the same was true of the pattern of Bone metastasis + others (excluding brain metastasis). While the pattern of metastases excluding bone and brain was the most common type in HER2-enriched and Triple Negative subtypes (68.5% and 65.6%, respectively). In addition, brain-related metastasis patterns accounted for a smaller proportion and were also more common in HER2-enriched and Triple Negative subtypes.

**Table 2:** Pattern of distant metastasis according to molecular subtypes.

	Total (%)	Luminal A (%)	Luminal B (%)	HER2-enriched (%)	Triple negative (%)	P value
Bone metastasis only	377 (16.0%)	83 (24.4%)	188 (18.9%)	48 (9.2%)	58 (11.5%)	<0.001
Metastases excluding bone and brain	1369 (58.1%)	158 (46.5%)	525 (52.8%)	356 (68.5%)	330 (65.6%)	
Bone metastasis + others (excluding brain metastasis)	530 (22.5%)	93 (27.4%)	249 (25.1%)	96 (18.5%)	92 (18.3%)	
Brain metastasis only	25 (1.1%)	1 (0.3%)	5 (0.5%)	6 (1.2%)	13 (2.6%)	
Brain metastasis + others	56 (2.4%)	5 (1.5%)	27 (2.7%)	14 (2.7%)	10 (2.0%)	

### Cumulative data of distant metastasis and molecular subtypes

Cumulative data of distant metastasis were shown in **Table 3**. Nearly half of ABC patients presented multiple metastases simultaneously. Bone was the most common site of distant metastasis (39.1%), and patients who were found to be positive in Luminal A and Luminal B accounted for 51.1% and 44.7% of patients who developed bone metastasis, respectively. Liver metastasis was more frequently observed in HER2-enriched ABC patients, accounting for 29.1%, and patients with HER2-enriched and Triple negative primarily presented lung metastasis more. Further, Triple Negative subtype was also more likely to have brain metastasis, but no statistically significant differences were found with other molecular subtypes.

**Table 3:** Cumulative frequency of distant metastasis sites according to molecular subtypes.

	Total (%)	Luminal A (%)	Luminal B (%)	HER2-enriched (%)	Triple negative (%)	P value
Number of distant metastasis (%)						
1	1249 (53.0%)	187 (55.0%)	528 (53.1%)	261 (50.2%)	273 (54.3%)	0.472
≥2	1109 (47.0%)	153 (45.0%)	467 (46.9%)	259 (49.8%)	230 (45.7%)	
Bone	935 (39.1%)	178 (51.1%)	452 (44.7%)	150 (28.7%)	155 (30.5%)	<0.001
Liver	569 (23.8%)	69 (19.8%)	265 (26.2%)	148 (29.1%)	87 (16.6%)	<0.001
Lung	729 (30.5%)	85 (24.4%)	299 (29.5%)	189 (36.1%)	156 (30.6%)	0.002
Brain	81 (3.4%)	6 (1.7%)	32 (3.2%)	20 (3.8%)	23 (4.5%)	0.145
visceral metastases*	1845 (77.1%)	275 (79.0%)	811 (80.1%)	369 (70.6%)	390 (76.6%)	<0.001

* including liver, lung, brain, adrenal gland and ovary, etc.

### Cumulative recurrence and metastasis rate

**Figure 1:**
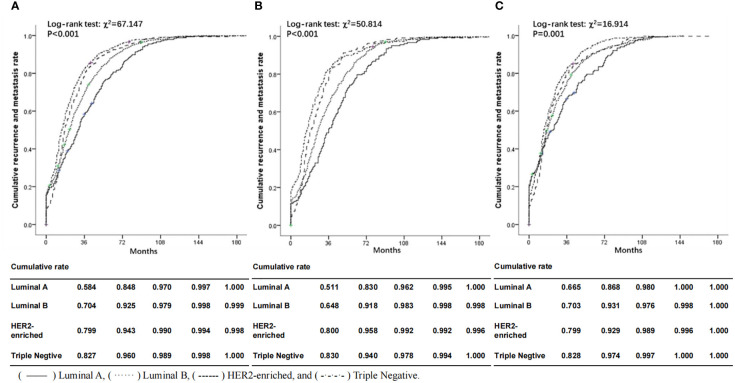
Comparisons of cumulative recurrence and metastasis rate according to molecular subtypes, overall **(A)** and in subgroups aged <45 **(B)** and ≥45 **(C)** at first diagnosis. Solid lines represent ABC patients with Luminal A subtype; dotted lines represent ABC patients with Luminal B subtype; dashed lines represent ABC patients with HER2-enriched subtype; dash-dot lines represent ABC patients with Triple Negative subtype. () Luminal A, () Luminal B, () HER2-enriched and () Triple Negative.

**Figure 1** illustrates the significant difference in cumulative recurrence and metastasis rate according to molecular subtypes. The median time of DFS was 29.0 months (range 0.1-38.7). Luminal B subtype had a poorer prognosis than Luminal A compared with Triple Negative, HER2-enriched subtype tended to spread more aggressively and was associated with higher cumulative recurrence and metastasis rates, whether from the whole or divided into two age subgroups (< 45 years and ≥45 years subgroups). It can be clearly seen from the curves that the cumulative recurrence and metastasis rates of ABC patients at 36 months of DFS were the most significant in ABC patients of all molecular types. The recurrence and metastasis rate of Triple Negative was the highest (82.7%), while that of Luminal A was the lowest, 58.4% of the tumors were recorded as recurrence and distant metastasis. Luminal B and HER2-enriched were between them. These differences still existed in the age subgroup analysis, especially in <45 years age group (Triple Negative: 83.0% versus. Luminal A: 51.1% at the 36-month time point). Both HER2-enriched and Triple Negative patients in younger age group showed higher cumulative recurrence and metastasis rates than those in older age group, but the Luminal subtype was the opposite.

### Association between molecular subtypes and distant metastasis sites

In Cox regression analyses, crude and fully adjusted HRs and 95% CIs for the associations between molecular subtypes and distant metastasis sites are shown in **Table 4**. ABC patients with HER2-enriched and Luminal B significantly increased the risk of liver metastasis (HR _HER2-enriched_ 2.27, 95% CI 1.65-3.14, HR _Luminal B_ 1.66, 95% CI 1.24–2.23), and the HRs remained statistically significant after extended adjustment to the model (HR _HER2-enriched_ 2.14, 95% CI 1.47-3.12, HR _Luminal B_ 1.40, 95% CI 1.01–1.96). Luminal B, HER2-enriched and Triple Negative were positively correlated to lung metastasis in the univariate as well as in the multivariate analyses. Further, these types of molecular subtypes also increased the risk of visceral metastasis by 23%, 46% and 87% respectively. In terms of brain metastasis, the results showed that triple Negative was associated with a significantly increased risk of brain metastasis (HR 3.99, 95% CI 1.58–10.12) and this association remained significant after full adjustment (HR 3.07, 95% CI 1.04–9.07). However, it made no statistical difference in the risk of bone metastasis of the three subtypes compared with Luminal A, whether by univariate or multivariate analyses.

**Table 4:** HRs and 95% CIs for the associations between molecular subtypes and the sites of distant metastasis in advanced breast cancer.

Sites of distant metastasis	Univariate analysis HR (95%CI)	P-value	Multivariate analysis* HR (95%CI)	P-value
Bone				
Luminal A	1.00		1.00	
Luminal B	1.17 (0.97-1.43)	0.109	1.06 (0.84-1.35)	0.628
HER2-enriched	0.99 (0.78-1.26)	0.968	0.94 (0.70-1.27)	0.686
Triple Negative	1.14 (0.89-1.46)	0.295	1.29 (0.96-1.73)	0.094
Liver				
Luminal A	1.00		1.00	
Luminal B	**1.66 (1.24-2.23)**	0.001	**1.40 (1.01-1.96)**	0.047
HER2-enriched	**2.27 (1.65-3.14)**	0.256	**2.14 (1.47-3.12)**	<0.001
Triple Negative	1.30 (0.63-2.67)	0.478	1.18 (0.90-1.53)	0.225
Lung				
Luminal A	1.00		1.00	
Luminal B	**1.74 (1.33-2.29)**	<0.001	**1.63 (1.18-2.24)**	0.003
HER2-enriched	**2.76 (2.07-3.68)**	<0.001	**2.46 (1.74-3.47)**	<0.001
Triple Negative	**2.65 (1.96-3.57)**	<0.001	**2.65 (1.86-3.78)**	<0.001
Viscus**				
Luminal A	1.00		1.00	
Luminal B	**1.35 (1.16-1.57)**	<0.001	**1.23 (1.02-1.48)**	0.032
HER2-enriched	**1.54 (1.29-1.83)**	<0.001	**1.46 (1.18-1.80)**	0.001
Triple Negative	**1.82 (1.53-2.16)**	<0.001	**1.87 (1.51-2.30)**	<0.001
Brain				
Luminal A	1.00		1.00	
Luminal B	1.87 (0.76-4.58)	0.171	1.72 (0.64-4.62)	0.285
HER2-enriched	**3.00 (1.18-7.63)**	0.021	1.94 (0.64-5.87)	0.242
Triple Negative	**3.99 (1.58-10.12)**	0.004	**3.07 (1.04-9.07)**	0.042

* Adjusted for age at diagnosis, tumor size, number of lymph node metastasis, menstrual status, education and family history.

** including liver, lung, brain, adrenal gland and ovary, etc.Bold text in this manuscript indicates statistical significance.

## Discussion

The complex biological behavior of breast cancer is determined by the heterogeneity of its intrinsic molecular phenotypes. There is marked variability in the time interval between treatment of the primary tumor and the occurrence of distant metastases, in the organs involved with distant metastases and in the response to systemic treatment in patients with metastatic breast cancer (16). Prior studies have reported that the general mechanism of tumor metastasis was composed of reciprocal interactions between tumor cells and host tissues, which included alterations in adhesion, proteolysis, invasion and angiogenesis (17, 18). However, only a few studies have described unique predilections and intrinsic association between molecular subtypes and distant metastasis sites among ABC patients, especially in Chinese native women. In this study, ABC patients in multiple centers in China were included, and the internal relationship between molecular subtypes and sites of distant metastasis was deeply discussed. In general, patients with Luminal A tended to develop bone metastasis and they had better survival outcomes than Luminal B and HER2-enriched with tendency of having visceral metastasis, Triple Negative with brain metastasis. Therefore, this study may fully represent the overall situation of distant metastasis pattern and prognosis of ABC patients at the national level. Further, these results lend support to the hypothesis that molecular subtypes predict the preferential sites of distant metastasis in ABC and may give valuable guidance to the formulation of personal targeted therapy strategy and the prediction of clinical prognosis.

Molecular subtypes of breast cancer, as an important complement to TNM staging, have the propensity to give rise to distant metastasis at specific sites. Recent publications had shown that newer molecular subtypes which used microarrays for gene expression analysis, was the best way to perform such molecular classification (19–21). However, such assays were mostly limited to the research laboratories and were not available for the routine clinical practice. Moreover, most of the archived clinical specimens were not suitable for this analysis. Therefore, the IHC-based classification systems are now still useful in clinical practice especially when performed by inexperienced centers, and have proven to be highly correlated with intrinsic classification using gene expression microarrays (22, 23). Also, there is significant agreement in the site of distant metastasis and outcome predictions for individual patients by these tests (24). For classification of molecular subtypes to be more helpful, ongoing efforts should be directed at standardization of current testing and development of more reliable and reproducible testing for ER/PR and Her2 gene expression (25).

Previous studies had demonstrated significant difference in the time of recurrence or distant metastasis within different molecular subtypes. In this study as expected, ABC patients with Luminal subtypes had relatively longer DFS and were prone to bone metastasis with good prognosis. While Triple Negative had a higher cumulative rate than other subtypes and more of them had a propensity of brain metastasis with the shortest DFS, highlighting a substantial clinical burden and unmet need for more effective treatment for these high-risk patients. These results indicated that distant metastasis of breast cancer was a non-random process, and ABC patients with different molecular subtypes have different distant metastasis mechanisms. In our study, we further observed the cumulative recurrence and metastasis rate of different subtypes on age subgroups, showing that the difference of cumulative rates were more significant in younger age group, manifested that the rates of HER2 and TNBC patients were higher than those of older age group, while rates were just opposite in Luminal types with good prognosis at the 3-year time point. Another retrospective study showed that DFS after adjuvant chemotherapy in younger patients with ER-positive tumors was significantly longer than patients with ER-negative disease (26). Colak et al. also described different gene expression profiles in breast tumors from younger women (27). They found lower expression of ER alpha and beta mRNA, with higher expression of HER2 and epidermal growth factor receptor (EGFR) compared to older patients. Moreover, elevated HER2 and EGFR were reported to be associated with distant metastasis especially brain metastasis (28). This finding could help to explain the disease course that we observed in young patients.

Characterization of breast cancer metastasis to bone has been most extensively studied since bone is the most common site of distant metastasis (29). With the arriving era of molecular profiling, it has become evident that bone metastasis was most common in the Luminal subtypes in one or more organ systems (30), as recapitulated in the current study. Bone metastasis is generally determined by spinal vein system which has the characteristics of no venous flap and low venous pressure, so cancer cells can be transferred to spinal vein prior to vena cava system, resulting in bone metastasis. Therefore, we speculate that this may be the primary reason for the highest rate of bone metastasis. In addition, there is increasing evidence that breast cancer cells have the ability to activate osteoclasts similar to that of normal breast gland epithelial cells during lactation, so breast cancer cells have the inherent characteristics of mutual benefit with bone tissue (31). Kroepil et al. also reported that SNAI1 was a zinc finger transcriptional repressor of CDH1, which encoded E-cadherin. Downregulation of E-cadherin was crucial to the dissemination and invasion of cancer cells, which might augment breast cancer metastasis into the bone (32). Moreover, the results of collective studies so far indicated that breast cancer with bone metastatic potential could be divided into two groups: those with bone metastasis only (ie, Luminal subtypes) and those with bone + other sites, the latter showed biological behavior similar to that of extra skeletal metastasis (33). This idea was supported in part by previous genetic analysis studies, but the underlying mechanism remained unclear. Further molecular studies are needed to explain the differences in biological behavior.

HER2 over-expression plays an important role in the proliferation, apoptosis, and angiogenesis of many solid tumors, so it is thought that the prognosis for patients with HER2 gene amplification should be poor. Some studies have shown that HER2-enriched was more likely to have multiple site and visceral metastases, among which liver metastasis was the most common (34). These findings were partly in keeping with the observations in our study. The observed liver-seeking characteristic of the HER2-enriched subtype was further strengthened by its significant association with liver metastasis in ABC patients. The mechanisms of liver metastasis in HER2-enriched ABC are complex and still largely unclear. Li et al. (35) reported that HER2 overexpression mediated a chemokine receptor, CXCR4 associated metastases. Therefore, we speculated that HER2 overexpression involved or promoted liver metastasis, but not peritoneal and lymph node metastases. Further study to clarify the mechanism of preferential liver metastases using HER2-enriched breast cancer cell lines will still be carried out.

To date, the association between breast cancer subtypes and lung metastasis in patients with breast cancer have been preliminarily identified (36). A tissue microarray analysis had showed HER2-enriched subtype, TNBC and the Luminal-HER2 all exhibited higher lung metastasis rates compared with Luminal A cancers (37). On multivariate analysis in this study, the probability of lung metastasis in HER2-enriched and Triple Negative subtypes was higher than Luminal type tumors. This was consistent with observations from previous studies describing that several signature genes was associated with increased lung metastasis risk (38, 39). These signature included EGFR, COX2, and the matrix metalloproteinases 1 (MMP1), CXCL1 and IDI1 which were highly expressed in the Triple Negative subtype and HER2-positive cancers. These genes collectively allow angiogenesis, tumor intravasation into the circulation, and breaching of lung capillaries by circulating tumor cells to seed the pulmonary parenchyma (40). This is the main reason why we infer the patients with the above molecular subtypes have the propensity of lung metastasis. In addition, miRNA profiling revealed that miR-629-3p was most commonly upregulated in both metastatic lesions and primary carcinomas in TNBC patients with lung metastasis compared with normal breast tissue (41). It should be hypothesized that miR-629-3p can create a specific microenvironment surrounding the metastasizing cells, necessary for invading and proliferating in lung issue. Therefore, accurate clinical testing of HER2, EGFR and other genomic makers may become necessary to provide complementary information for predicting the possibility of lung metastasis.

It is obvious from studies discussed that Triple Negative is a subtype of breast cancer that is associated with high risk of developing brain metastases, with an associated subsequent poor survival outcome (42–45). It has been reported that breast cancer, especially metastatic breast cancer, had a higher rate of brain metastasis, which was considered secondary because many new therapeutic drugs may not be able to penetrate the blood-brain barrier (46). In agreement with prior studies, brain metastasis was infrequent as the first site of distant metastasis in the present series. However, an increasing rate of brain metastasis was reported in other studies (47–49), and we also found a strong association between Triple Negative and elevated brain metastasis risk in the Cox regression model. This expected result has meaningful implications for communication and care decisions and is worth further molecular investigation. Furthermore, the median DFS of all patients with Triple Negative ABC in the present study was less than 2 years (18.5 months) and the 3-year cumulative recurrence and metastasis rate was 82.7%. The results were comparable to those by Lin et al. (50) in which median DFS of Triple Negative breast cancer was 19.9 months and 75% of metastases occurred within 3 years of the diagnosis of breast cancer. The reason may be related to the special biological characteristics and gene expression in different molecular subtypes. Previous studies have suggested that cell chemokines (CXCR4/CXCL12), PTHrP and NF-κB were important molecular markers affecting the metastasis rate of patients (51). The results of genome studies (52–54) showed that these markers were overexpressed in Triple Negative subtypes, suggesting that it was reasonable for Triple Negative breast cancer patients to have a higher distant metastasis rate. Therefore, exploring new therapeutic targets will become a new hotspot in Triple Negative breast cancer research.

Adding to the previous literature, the current study could provide important evidence for decision-makers. From the perspective of physicians, molecular subtypes can be used as an important tool for prognosis assessment of ABC patients, and it is an important supplement in assessing the time and site distribution of postoperative distant metastasis, developing targeted preventive therapeutic schedules for patients, and contributing to personalized screening during postoperative follow-up. For ABC patients, the difference in the distant metastasis pattern and overall prognosis of different molecular subtypes can provide guidance to choosing a reasonable and cost-effective approach for treatment, so as to improve the quality of life and promote rehabilitation. While for health policy maker, understanding the burden of different molecular subtypes of ABC could help in optimizing resource allocation for those who in greater need of advanced treatment.

The present study exhibits several strengths. Firstly, our study was the first multi-center hospital-based clinical epidemiological investigations to explore the characteristics of ABC for women in China. All the data was collected from 21 tertiary hospitals in seven geographical regions, representing different cancer burden levels, diagnoses, and treatments. Covering different geographic regions in China would be more informative and less subject to practices specific to individual hospitals which can provided important insights about real-world clinical outcomes for ABC patients from a large nationwide sample. Secondly, convenience sampling was adopted, and the sample size was allocated according to the month to reduce the bias caused by the time of initial diagnosis. In addition, a full review of the medical records permitted a check of the detailed documented metastasis sites, thereby providing a more apparent pattern as well as cumulative frequency of distant metastasis sites.

However, several potential limitations of this study should be considered inherent to hospital-based retrospective study. Firstly, the results might be subject to selection bias if confounding factors were not identified or adjusted for in the analyses. In the current study, we adjusted for confounders such as age at diagnosis, tumor size, menstrual status and family history which were thought to have the potential to affect the prognostic outcomes. In addition, molecular typing characteristics still need to be evaluated in conjunction with conventional or established gold criteria such as imaging and pathology.Secondly, the consistency of molecular typing between relapsed and metastatic tumor and primary tumor was not examined in this study, as there have been reported the rate of subtype conversion was 0% in basal-like tumors, 23.1% in HER2-enriched tumors, 30.0% in Luminal B tumors, and 55.3% in Luminal A tumors (55). The main reason was that the subtypes of some distant sites in medical records were unknown. However, given the clinical consequences of discordance, it urgently requires to deeply understand the differences between primary and metastatic tumors and develops the proper management of cancer patients in future studies. Thirdly, modern chemotherapy and adjuvant trastuzumab have reduced metastasis risk in HER2-positive disease (56, 57). In this study, adjuvant chemotherapy was only prescribed for 11.5% and 18.5% of ABC patients with Luminal B and HER2-enriched tumors. Therefore, it may be plausible if we consider that the results may be underestimated when discussing the distant metastasis risk with HER2-positive subtypes. Further studies to clarify this point are warranted. Due to these limitations and confounders, the study results need to be viewed with some caution, although several of the findings are well in line with prior clinical studies or with experimental data.

## Conclusions

In conclusion, this study provided data of ABC patients to clearly show that molecular subtypes were significant different in metastatic behavior with regard to the sites of distant metastasis as well as recurrence and metastasis rate, suggesting that molecular subtypes could predict the preferential site of distant metastasis in female ABC patients. Despite improving breast cancer outcomes, distant metastasis remains common and incurable, especially for the ABC patients. These observations could potentially be used in determining the appropriate schemes for follow-up of ABC patients and hopefully shed light on the development of effective surveillance strategies and targeted therapies. Therefore, future studies are warranted and can ultimately lead to the tailoring of individualized comprehensive treatment fields based on molecular subtypes combined with the conventional clinicopathologic characteristics.

## Data availability statement

The datasets used and/or analysed during the current study are available from the corresponding author on reasonable request. Requests to access the datasets should be directed to You-lin Qiao, qiaoy@cicams.ac.cn.

## Ethics statement

The studies involving human participants were reviewed and approved by Cancer Hospital, Chinese Academy of Medical Sciences. Written informed consent for participation was not required for this study in accordance with the national legislation and the institutional requirements.

## Author contributions

Y-LQ, J-YW, and B-HX had access to all of the data in the study and take responsibility for the integrity of the data and the accuracy of the data analysis. Y-LQ and B-HX participated in the study concept and design. SZ acquired and analyzed data. J-HF, SZ, and HY were responsible for interpreting the data. J-HF, SZ, and HY took the lead in drafting the report. The other co-authors collected the data from the corresponding study hospital. Every author participated in editing and finalization. The work reported in the paper has been performed by the authors, unless clearly specified in the text. All authors contributed to the article and approved the submitted version.
